# Supplementary material for: Are carbon emissions trading and green financial instruments synergistic? -Comprehensive quantitative research based on content analysis
Source: PLoS One. 2024 Mar 7;19(3):e0298601. doi: 10.1371/journal.pone.0298601 (PMC10919601; doi:10.1371/journal.pone.0298601)
Supplement: S1 Data — (ZIP) [file pone.0298601.s001.zip › Data/地级市Z_F工作报告.zip!/┴╔─■/┼╠╜⌡╩╨/└┤╫╘╣1⁄2╓┌║┼í╛┬φ┐╦╩2╛▌═°í┐_2018.docx]

各位代表：

　　现在，我代表市人民政府，向大会报告工作，请予审议，并请市政协委员和其他列席同志提出意见。

　　一、过去四年工作回顾

　　市七届人大一次会议以来的四年，是盘锦发展进程中极不平凡的四年。在省委、省政府和市委的坚强领导下，我们认真学习贯彻习近平新时代中国特色社会主义思想，坚持发展第一要务不动摇，适应把握引领经济发展新常态，持之以恒贯彻落实新发展理念和“四个着力”“三个推进”，抢抓中央新一轮振兴东北老工业基地战略机遇，迎难而上，开拓进取，“十二五”圆满收官，“十三五”顺利实施，全市经济平稳健康发展、社会事业全面进步、群众生活明显改善，盘锦开启了建设国际化中等发达城市的新征程。

　　综合实力和竞争力显著增强。坚持把稳增长作为首要任务，切实转变发展方式，推动科学发展，经济运行稳中向好。2013年以来，各项主要经济指标实现总量壮大和位次前移。2017年，预计全市地区生产总值完成1075亿元，固定资产投资完成603亿元、在全省位次由第五位升至第三位，一般公共预算收入完成118.9亿元、由第五位升至第四位，规模以上工业增加值完成398亿元、由第六位升至第三位，社会消费品零售总额完成393亿元、位次前移两位，城乡居民人均可支配收入分别以3.66万元、1.59万元稳居全省第三和第二位。辽河油田稳产千万吨，华锦集团等中央企业在改革创新发展中扭亏为盈、做优做强；北燃公司、兴隆集团等一批民营骨干企业不断成长壮大，年均新注册市场主体2万户，各种所有制经济、大中小微企业竞相发展，成为稳增长的压舱石。兵器集团精细化工及原料工程顺利奠基，实施宝来石化等10亿元以上重大项目52个，世界500强、行业100强企业达到10户，累计开工建设亿元以上项目768个、竣工512个。在国务院第三次大督查中，盘锦成为全省唯一受到通报表扬的地级市。省政府绩效考核成绩始终名列前茅，盘锦成为东北地区最具发展潜力的城市之一。

　　结构调整取得重要进展。“三去一降一补”全面落实，关停小化工、小炼油等不达标企业347家，房地产库存去化周期保持在合理区间，提升非信贷融资比例促进了企业杠杆率有效降低，落实“营改增”、取消部分行政事业性收费、直购电交易等政策为企业减负近10亿元，共争取到99.7亿元国家专项建设基金、104.8亿元企业债券、408.3亿元补助资金，推出18个重点领域标准化改革并取得实质性进展。制定实施《中国制造2025盘锦行动纲要》，工业主导产业向高端化、集群化方向发展，世界级石化及精细化工产业基地建设实现新突破，国内领先的能源装备制造产业基地建设取得新进展。高质量完成服务业供给侧结构性改革三年行动计划，明厨亮灶和集贸市场、商网门脸等改造提升工程实现传统服务业重塑再造，临港粮食物流加工、快递物流、电子商务等现代服务业蓬勃兴起；红海滩廊道通过国家5A级景区质量评定，“红海滩+”旅游品牌叫响全国，特色乡村游备受青睐，全域旅游发展态势初步形成。设施农业、认养农业比重逐年提升，市级以上农业产业化龙头企业发展到70家，盘锦大米品牌价值跃居全国第二位，河蟹出口创汇位列全国地级市之首，粮食生产连续十四年大丰收。国家重点实验室、院士工作站、产业技术研究院的组建和引进实现重大突破，中蓝电子等一批高新技术和小巨人企业迅速崛起，我市高新区以省级考评第一的成绩向国家级高新区迈进。

　　全面深化改革实现重点突破。累计推出100余项改革举措，取消和下放市本级行政职权1131项。市级权力全面赋予县区、经济区，推行容缺审批、企业承诺、全程代办审批模式及“二十六证合一、一照一码”改革使审批时限压缩了近三分之二，“双随机、一公开”监管全面推行，营商环境综合整治行动成效明显，国家工商总局在盘锦设立商标受理窗口，我市在全省首获县区外商投资企业登记管理权限。夯实“1+8”改革促进基层治理体系完善和治理能力提升，市县镇村四级政务服务网全面构建形成。精简机构编制、事业单位转企改制使财政供养人员只减不增。“1+6+2”国有集团公司组建运营，“四资联动”改革向纵深推进，农垦改革试点全面铺开，央企“三供一业”分离移交完成阶段性任务。市商业银行获批更名为盘锦银行，设立产业创业投资引导基金、信贷风险补偿基金、应急转贷资金积极改善投融资环境。集中推出财政零基预算、强化公共资源交易管理、“五险统一”征缴、个体零散税源征管等开源节流举措。建成全国一流的智慧城市管理运营中心，实现政务服务和社会治理资源信息的高效集成联动，在全国率先实现社会治理全域网格化数字化。

　　沿海开发开放迈出新步伐。扩大全域全面对外开放，实际利用外资累计完成11亿美元，连续四年位居全省前列；进出口总额累计完成61.9亿美元，年均增长34.7%。辽东湾新区港产城联动发展实现新跨越，主要经济指标位居国家级开发区中上游，成为国家循环化改造重点支持园区。一批现代石化骨干企业集聚发展，中储粮等一批国内顶尖粮食物流加工企业相继入驻。盘锦港累计建成21个5万吨级以上泊位，港口通过能力提高到6000万吨；疏港铁路、沈盘铁路和疏港高速、沈西工业大道连接线竣工通车，以港口为核心的集疏运体系快速形成；成功获批国家一类口岸，保税物流中心封关运行，开辟集装箱班轮航线13条，挂牌内陆干港6个，盘锦成为东北及蒙东地区“一带一路”战略三条国际通道的重要节点。新区复制落地49项自贸区改革试点政策，在全省率先建立企业投资项目承诺制，实现市以下行政事业“零收费”。市民服务中心、六大文化场馆、八大创业孵化基地以及学校、幼儿园等公共服务设施相继竣工，城市功能进一步完善，树立起现代化港口城市新形象。

　　城乡一体化发展呈现新面貌。全域城市化迈出坚实步伐，城镇化率高于全省平均水平5个百分点。全面开展美丽乡村建设，305个行政村全部建成美丽乡村并持续提档升级，我市成为全省唯一的全域美丽乡村建设试点市。黑色路面全覆盖，农村燃气户户通，9万多农户用上清洁能源取暖，垃圾分类全面推开，城乡一体的大环卫体系投入运营，105个小型污水处理设施基本建成，村村拥有标准化卫生室、连锁超市，涌现出一批特色旅游村、精品民宿村，赵圈河镇、胡家镇跻身国家级特色小镇。大洼撤县设区，城市总体规划获得国务院批复，“多规合一”平台启动运行。公路铁路主干路网、城乡主次干道全面完善，实施东外环、中新线、林丰路大桥等50项重点工程，形成了“四铁四高”、“一环七纵六横”的大交通格局。大力开展国际化城市交通提升行动，打造形成国际化交通样板路。全域客运公交一体化改造扎实推进，国家“十三五”首批公交都市示范市创建工作启动实施。完成双台子河闸除险加固工程，新建和改造城市排水泵站15座、排水管网153公里，盘锦连续三年获得省“大禹杯”。大伙房水库输水工程让城乡居民喝上了优质放心水。大力度实施强弱电入地及绿化园艺化、景观亮化、楼体美化工程，新建改扩建城市公园8座，植树造林1730万株，城市绿地覆盖率达到38.3%。我市城乡面貌日新月异。

　　人民群众提前过上了小康生活。践行以人民为中心的发展思想取得实效，全市财政支出的75%用于民生改善。城镇新增就业人数年均超过2万人，城镇登记失业率保持全省最低。开展以辽河口三年攻坚为重点的精准扶贫脱贫，贫困人口保持动态为零。覆盖全民的社保体系基本建立，城乡基本养老保险制度实现全覆盖，医疗工伤生育保险实现市级统筹，城乡低保救助标准分别提高23%和26%。在全省率先基本完成城市棚户区和农垦职工危旧房改造任务，弃管楼宇整治让20余万居民从中受益。教育质量稳步提升，3个县区通过国家义务教育发展基本均衡验收，大连理工大学盘锦校区在内涵式发展中凸显了人才和创新支撑优势。制定实施《“健康盘锦2030”行动纲要》，建成四大远程会诊中心和四级联动的医疗卫生服务体系，城乡居民医疗保险实行按疾病分组支付，率先实现新农合报销村级即时结算。城乡社区养老服务设施覆盖率全省最高，盘锦成为中央财政支持的居家和社区养老服务改革试点市。强化食品药品安全监管，学校幼儿园食堂实行食材统一配送，盘锦连续8年食品药品安全零事故。高水平举办三届“红马”赛，盘锦健儿在世界、洲际和国家级体育比赛中共获得11枚金牌。充分挖掘整理辽河口地域文化，镇村两级综合文化中心全面建成，国家公共文化服务体系示范区创建顺利通过中期检查。国防动员、边海防、人防工作不断加强，五次蝉联全国双拥模范城。

　　生态文明建设跃上新台阶。制定实施《盘锦市国家生态文明建设示范市规划》，大洼区成为全省唯一的国家首批生态文明建设示范区。加大湿地保护和修复力度，实现退养还滩2.3万亩、退耕还湿1万亩，保护区内退出油田生产设施524处，芦苇湿地补水面积达到52万亩，辽河口湿地位列中国十大最美湿地之首。综合开展能源结构调整、重点企业提标改造、小锅炉和黄标车淘汰等工作，空气质量综合指数全省第三。全面建立“河（湖）长制”，主要河流水质持续改善，螃蟹沟（六零河）改造工程使我市成为全省率先完成黑臭水体整治的城市。全面完成禁养区内畜禽养殖企业关闭搬迁任务，规模养殖场废弃物处理达到无害化标准。完成永久基本农田划定任务，耕地保护工作始终处于全省领先地位。倡导绿色低碳生产生活方式，公共机构节能考核连续三年位居全省前列。中央环保督察、国家海洋专项督察期间，我市信访案件全省最少，湿地修复和农村环境治理得到中央环保督察组的高度肯定。盘锦的天更蓝、水更清、空气更清新，正以更加坚实的步伐走向生态文明新时代。

　　政府治理能力得到全面提升。坚持科学、依法、民主行政，充分发挥了保持经济稳定增长、优化公共服务、保障公平竞争等职能作用。坚持市政府党组向市委请示报告工作制度，坚决贯彻落实市委重大决策部署。自觉接受市人大法律监督、工作监督和市政协民主监督，市人大代表建议450件、市政协提案903件全部办复，满意率均达100%。建立重大决策合法性审查制度，起草并提请市人大常委会审议通过地方性法规2部。积极听取和采纳民主党派、工商联和无党派人士的意见，全面为工会、共青团、妇联等群团组织开展工作创造条件。健全立体化社会治安防控体系，严厉打击各类违法犯罪行为，相继侦破中央领导批示的特大骗取贷款、公安部督办的特大跨省制贩毒等要案，我市成为最有安全感的城市之一。常态化开展安全稳定工作调度评估，连续10年被省政府评为安全生产先进市，连续多年信访工作综合考核全省第一，连续两届捧得国家综治最高奖项“长安杯”。“六五”普法圆满完成并荣获全国法治宣传教育先进市，“七五”普法顺利实施。行政监察不断加强，审计监督实现全覆盖，深入开展行政执法、项目招投标、工程建设以及损害群众利益等领域专项整治，一批违法违纪案件得到查处。严格落实中央八项规定精神，大力整治“四风”问题，完成行政机关公务用车改革，“三公”经费实现四连降。

　　民族宗教、外事侨务、海关、边检、检验检疫、海事、气象地震、统计档案、邮政通信、红十字会、残联、地方志等各项工作都取得了新成绩。对口援助工作取得积极成果。

　　各位代表，四年攻坚克难，四年接力奋斗，我们积极争取在各个领域站上国家平台、在各个方面抢占发展先机，在激烈的竞争中赢得了优势和主动，办成了许多过去想办而没有办成的大事。我们举全市之力推进“多城联创”，盘锦荣膺全国文明城市，获批国家卫生城市、国家园林城市，蝉联全国社会治安综合治理优秀市，共争取到26个国字号试点（示范）。这一系列殊荣成为盘锦的一张张亮丽名片，必将对我市经济社会发展产生重大而深远的影响。

　　与此同时，我们也清醒地认识到，当前我市在发展中还存在一些问题。结构性矛盾仍较突出，民营经济发展不充分，现代服务业发展水平不高，经济外向依存度仍然较低，镇域经济实力不强，村级集体经济薄弱；体制机制需要进一步完善，创新不足，人才短缺，软环境建设还需进一步加大力度；个别干部懒政怠政、推诿扯皮，不作为、滥作为的行为时有发生。对此，我们要采取更加有力的措施，认真加以解决。

　　各位代表，过去四年的成绩来之不易。面对世界经济复苏乏力、我国经济发展新旧动能转换的大环境，在全省经济艰难筑底企稳、下行压力持续加大的背景下，全市人民戮力同心、砥砺奋进，创造了改革开放和国际化现代化建设的辉煌业绩。在此，我代表市政府，向奋战在各条战线上的人大代表、政协委员、广大干部，向各民主党派、各人民团体和各界人士，向驻盘部队、武警官兵，向辽河油田、华锦集团、辽河石化等驻盘中省直单位，向离退休老同志以及所有关心、支持和参与盘锦建设发展的同志们、朋友们，表示衷心的感谢！

　　回顾四年的光辉历程，用市委七届七次全会提出的“四个坚持”来审视衡量政府工作，关键在于我们始终牢固树立“四个意识”，在思想上政治上行动上同以习近平同志为核心的党中央保持高度一致，确保党中央国务院、省委省政府和市委的各项决策部署不折不扣落到实处。关键在于我们始终坚持发展第一要务，坚定不移贯彻落实新发展理念，持之以恒推进实施“四个着力”“三个推进”的全链条部署，确保各项工作始终走在全省乃至全国前列。关键在于我们始终坚持用国际化视野谋划发展、站上国家平台推动建设，加快实施《国际化中等发达城市战略规划纲要》，做到一张蓝图绘到底。关键在于我们始终聚焦“多城联创”加强和改进全方位各领域工作，统筹推进经济、政治、文化、社会、生态、党建等各项事业协调发展、全面进步。关键在于我们始终坚持在发展中保障和改善民生，每年集中推出一批惠民举措，全市人民获得感、幸福感、安全感、自豪感显著提升。关键在于我们始终聚焦履行“一岗双责”推进干部作风转变，形成项目化、日历化抓落实的一整套工作模式和推进体系，引导党员干部谋实招、办实事、求实效，创造出经得起历史、实践和人民检验的业绩。

　　二、今后五年的总体安排

　　各位代表，今后五年，是全面完成“十三五”规划、制定实施“十四五”规划的历史交汇期，是滚石上山、爬坡过坎的攻坚期，也是我市发展的重大战略机遇期。党的十九大明确了新时代中国特色社会主义发展的战略安排，省委、省政府作出“一带五基地”建设和实施“五大区域发展战略”的决策部署，为盘锦发展指明了前进方向。

　　市委七届七次全会审议通过了《中共盘锦市委关于高举习近平新时代中国特色社会主义思想伟大旗帜深入学习贯彻党的十九大精神加快建设国际化中等发达城市的实施意见》，进一步描绘了全面建设现代化、都市化、国际化中等发达城市的宏伟蓝图，进一步明晰了“三步走”的时间表、路线图、任务书。到二○二○年，我们要在国家全面建成小康社会、新一轮东北振兴取得重大进展的背景下，全面打好建设国际化中等发达城市的各方面基础，进入现代化、都市化、国际化发展轨道。到二○三○年，我们要在国家现代化建设取得重大进展、辽宁实现全面振兴的背景下，城乡现代化基本实现，全域都市化基本完成，国际化中等发达城市基本成型。到二○四○年，我们要在基本实现现代化、都市化、国际化的基础上，全面建成全域体现现代化水平、具有都市化特征的国际化中等发达城市。

　　按照《实施意见》的总体安排，围绕实现“三步走”战略目标，今后五年政府工作的指导思想是：全面贯彻党的十九大精神，以习近平新时代中国特色社会主义思想为指导，坚持加强党对经济工作的集中统一领导，紧扣社会主要矛盾变化，按照高质量发展的要求，统筹推进“五位一体”总体布局，协调推进“四个全面”战略布局，全面落实新发展理念和“四个着力”“三个推进”，按照省委省政府“一带五基地”建设和“五大区域发展战略”决策部署，以供给侧结构性改革为主线，扎实做好稳增长、促改革、调结构、惠民生、防风险各项工作，大力推进改革开放，推动质量变革、效率变革、动力变革，在打好防范化解重大风险、精准脱贫、污染防治的攻坚战方面取得扎实进展，促进经济社会持续健康发展，加快盘锦现代化、都市化、国际化进程，全面建设全域体现现代化水平、具有都市化特征的国际化中等发达城市。

　　各位代表，中国特色社会主义进入新时代，盘锦发展进入新境界。综合分析研判“经济发展由量变向质变转换、城市建设由粗放向精细转型、人民生活由基本需求向美好生活需要转变”的阶段性特征，我们总体上要努力实现“四个高于”、“三个同步”的发展预期：地区生产总值、一般公共预算收入、固定资产投资、规模以上工业增加值年均增速高于全国平均水平1个百分点，高于全省平均水平；城乡居民收入与地区生产总值基本同步增长，生态环境质量与经济质量效益同步改善，社会事业进步与经济发展水平同步提高。

　　做好未来五年工作，我们要按照市委七届七次全会提出的“七个必须”要求，牢牢把握以下几个方面。

　　（一）坚持新发展理念不动摇。发展是解决盘锦一切问题的基础和关键。我们要坚定不移把发展作为第一要务，坚持不懈贯彻新发展理念和“四个着力”“三个推进”，统筹县区、经济区、园区，兼顾国有与民营、存量与增量、速度与质量、当前与长远，加快形成推动高质量发展的指标体系、政策体系、标准体系、统计体系、绩效评价、政绩考核，推动新型工业化、信息化、城镇化、农业现代化同步发展，促进经济社会持续健康发展。

　　（二）坚持质量第一效益优先。盘锦正处在转变发展方式、优化经济结构、转换增长动力的攻关阶段。我们要坚定不移把发展的着力点放在实体经济上，深化“三去一降一补”，重点在“破”“立”“降”上下功夫，优化存量资源配置，扩大优质增量供给，做到投资有回报、产品有市场、企业有利润、员工有收入、政府有税收、环境有改善。

　　（三）坚持完善国际化市场化体制机制。改革开放是建设国际化中等发达城市的“关键一招”。我们要坚定不移构建形成系统完备、科学规范、运行有效的一整套制度体系和开放型经济体制，落实《沿海经济带三年行动计划》，加快建设世界级石化及精细化工产业基地，着力打造亿吨大港，基本建成东北亚国际能源物流中心、国家级粮食物流中心，促进域内外要素有序自由流动、资源高效配置、市场深度融合，加快培育形成现代化产业体系和竞争新优势。

　　（四）坚持实施乡村振兴战略。“三农”工作是盘锦实现国际化的决定性因素。我们要坚定不移按照产业兴旺、生态宜居、乡风文明、治理有效、生活富裕的总要求，落实抓乡村振兴责任制和“四优先”工作导向，大力实施《县域经济发展三年行动计划》，力争盘山县冲进全国百强，县域经济年均增幅高于全省平均水平，重塑城乡关系，加速城乡融合，实现农业强、农村美、农民富，朝着全域都市化目标迈进。

　　（五）坚持人与自然和谐共生建设美丽盘锦。绿苇红滩就是盘锦的金山银山。我们要坚定不移走生产发展、生活富裕、生态良好的道路，形成节约资源和保护环境的空间格局、产业结构、生产方式、生活方式，保护好湿地生态这一自然特色，建成国家生态文明建设示范市，打造世界级生态休闲旅游目的地。

　　（六）坚持以人民为中心的发展思想。增进民生福祉是发展的根本目的。我们要坚定不移把人民对美好生活的向往作为奋斗目标，在发展中补齐民生短板、促进社会公平正义，在幼有所育、学有所教、劳有所得、病有所医、老有所养、住有所居、弱有所扶上不断取得新进展，让全市父老乡亲的生活一年更比一年好。

　　以上六个方面，是我们做好今后五年工作的重要遵循。必须长期坚持，在各项工作中全面贯彻落实。

　　三、2018年重点工作

　　今年是贯彻党的十九大精神的开局之年，是改革开放40周年，是决胜全面建成小康社会、实施“十三五”规划、建设国际化中等发达城市的关键一年。做好今年的政府工作，意义十分重大。我们要全面落实中央经济工作会议、省委十二届五次和六次全会精神，按照市委七届七次全会暨市委经济工作会议的安排部署，坚持以党的十九大精神和习近平新时代中国特色社会主义思想为引领，聚焦发展不平衡不充分的八个方面问题，实施沿海开发开放、县域经济发展、乡村振兴“三大战略”，全力做好十项重点工作。力争地区生产总值增长7.5%，规模以上工业增加值增长8%，固定资产投资增长8%，一般公共预算收入增长12%，社会消费品零售总额增长6%。确保主要经济指标增幅高于全国平均水平1个百分点，绝大多数指标增幅超过全省平均水平，城乡居民收入增长与经济增长基本同步。

　　第一，毫不动摇抓项目稳增长

　　扎实开展项目年活动。健全完善重点项目领导包保责任制，做到市县（区）镇（街）村和园区一起抓、国资民资外资一齐上、一二三产业融合发展，筑牢以项目为核心的鲜明导向，促进重点项目尽快落地建设投产。确保兵器集团精细化工及原料工程项目开工建设，力争全年亿元以上项目新开工105个、竣工105个，续建项目复工率达到90%以上。

　　抓住用好战略机遇。深入贯彻落实东北老工业基地振兴和辽宁沿海经济带开发开放战略，争取更多的政策、项目、资金在盘落地见效，争取辽东湾新区纳入长兴岛国家石化产业基地拓展区，积极承接北京、上海地区的产业转移。加强与江苏省无锡市对口合作，努力在精细化工产业和农产品销售合作等方面实现突破。

　　加大招商选资力度。紧盯重点国家和地区的重点产业走出去登门招商，持续开展“看盘锦变化、助家乡发展”请进来活动，精心包装项目实施精准招商，推进产业链招商、点对点招商、以商招商、全民招商，切实做到大招商、招大商、招好商。全年签约注册项目力争超过200个，其中亿元以上项目100个、5亿元以上项目20个、10亿元以上项目5个，实际利用外资、到位内资分别增长15%以上。

　　着力抓好企业运行。坚持分类指导、因企施策，提高土地、资金、人才等要素保障水平，推动现有优势企业提高市场竞争力，帮助困难企业解决实际问题，引导有特色有潜力的企业转型升级、扩能改造，着力激发各类市场主体活力。

　　大力发展民营经济。开展国家民营经济发展改革示范工作，全面实施并不断完善市场准入负面清单制度，破除歧视性限制和各种隐性障碍，加快构建亲清新型政商关系。落实保护产权政策，依法处理社会反映强烈的产权纠纷案件。落实促进民营经济发展“十条”政策，规范推进PPP合作模式，实施大做强、小升规、个转企、限下升限上四项工程，促进民营大中小微企业竞相发展。

　　打造国际化营商环境。完善政府守信践诺机制，打造“诚信盘锦”。深入实施优化政务环境等八个方面专项整治，开展投资环境治理“十项工程”，实施全流程、全周期“妈妈式”服务，建设全省营商环境最佳城市。

　　第二，坚定不移抓供给侧结构性改革

　　落实“三去一降一补”。重点在破除无效供给、培育新动能、降低实体经济成本上下功夫，加快结构调整步伐。加大“散乱污”企业关停并转力度，坚决淘汰不达标落后产能。房地产库存去化周期保持在合理区间。加大股权融资力度，引导有条件的企业挂牌上市。落实系列减税降费政策，切实为企业降成本减负担。围绕公共服务、基础设施、科技创新、资源环境、产业产品等重点领域，卓有成效解决不平衡不充分问题。

　　推进新型工业化。深入实施《中国制造2025盘锦行动纲要》，促进工业提质增效。聚焦石化及精细化工这个当家产业，狠抓大项目建设和产业链延伸，促进石化产业高新化、精细化、绿色化。引导传统能源装备制造业转型升级，推动忠旺铝制品以及长春石化锂电池铜箔等新能源新材料产业发展。全力支持中蓝电子光学产业基地、盘锦精细化工孵化基地等项目加快建设，引领新兴产业实现重点突破。实施工业强基工程、智能制造工程，突出抓好15个重大技术改造项目、30个智能制造项目建设，工业投资增长11%。

　　加快服务业提档升级。推进国家产融合作试点，推动盘锦银行引入战略投资者，加快组建盘锦农商行，新引进金融机构3家，实现金融服务实体经济能力和自身经营效益的双提升。发挥辽东湾临港物流园和盘山东北电商快递物流园的牵动作用，发展现代物流产业。积极发展会展经济，争取一批世界级展会和国际国内重大会议、重要赛事落户盘锦。着眼为骨干企业配套，培育壮大研发设计、检验检测等生产性服务业。实施服务业标准化提升工程，推动电子商务与传统服务业融合发展。引进健康养老、医学美容等新兴业态。

　　树立全域旅游新形象。实施“2019相聚盘锦”迎客入盘行动计划，丰富“红海滩+”、“红马”赛事、冰凌穿越旅游产品体系。确保红海滩国家风景廊道晋升为5A级景区，抓好玉桂园等重点项目建设，推动士林不夜城等项目投入运营，依托旅游特色村、民族文化村培育一批乡村A级景区，实现旅游资源整合、线路优化、效益提升，全力争创国家全域旅游示范市。全市旅游业总收入增长12%以上。

　　加快发展现代农业。落实粮食安全责任制，确保国家粮食安全。坚持质量兴农、绿色兴农，发挥5个国家地理标志保护产品的引领作用，大力推广“一水三养、一地四收”模式，新增稻蟹综合种养10万亩、设施农业4万亩，建设高标准农田10万亩，发展认养农业35万亩。突出抓好高升省级农产品加工示范集聚区建设，打造辐射东北的农产品加工集聚区。

　　强化质量建设。深入推进质量提升专项行动，鼓励企业争创中国质量奖和省长、市长质量奖，引导产业园区创建品牌示范区。继续推进18个重点领域标准化改革建设。积极争创国家农产品质量安全市。

　　第三，持之以恒抓重点领域改革

　　加快转变政府职能。深化“放管服”改革，实施“多评合一、多规合一、多图联审”，拓展延伸“多证合一”，推进辽东湾新区“证照分离”改革，做到企业登记全程电子化。全面推进“双随机、一公开”监管。开展政务审批服务标准化国家试点，实现四级政务审批服务机构网上审批、跨域服务。稳步推进政府机构改革，深化事业单位分类改革。

　　深化国企国资改革。完善国企国资改革方案，统筹推进“四资联动”改革、国企三项制度改革、混合所有制改革，促进国有资本做强做优做大。推动“1+6+2”集团公司完善法人治理结构、提升管理水平，力争各集团利润总额增长10%，资产负债率控制在合理水平。

　　推进央地深度合作。全力支持辽河油田千万吨稳产，支持华锦集团、辽河石化增产增效。完成6户驻盘央企“三供一业”分离移交任务。鼓励央企优先采购地产机械设备、仪器仪表等配套产品，地方要顺应央企需求引进培育发展关联产业。发挥央企的主导作用，吸引域外配套服务商来盘注册企业、投资兴业。

　　抓好农垦改革试点。全面落实《农垦改革发展综合试点实施方案》，推进农垦集团及农场有限公司市场化运营，完成国家深化农垦改革专项试点任务，为全省农垦改革树立样板。

　　深化财税体制改革。坚持先生活后生产原则，加强预算执行管理，调整优化财政支出结构，确保对重点领域和项目的支持力度。实施国地税业务一窗口通办、地税与不动产登记一体化联办，全面推行个体零散税源社会化管理，开征环保税。加强公共资源交易精细化管理。

　　强化风险管控。做好重点领域风险防范和处置，加强薄弱环节监管制度建设。继续做好政府存量债务置换工作，优化政府性债务结构。落实金融稳定协调合作机制，防范化解金融风险。确保养老金按时足额发放。

　　第四，多措并举抓全域全面开放

　　增强港口综合功能。抓住香港招商局集团港口资源整合契机，进军全省战略发展重点港区。抓好11个5万吨级以上泊位建设，推进10万吨级航道工程开工，港口通过能力达到7000万吨。建立辽东湾新区与辽宁自贸区协同合作机制，积极申建综合保税区、国家进境粮食指定口岸。推进石化产品交易中心、中储粮东北粮食运营中心等交易平台建设，推广应用国际贸易“单一窗口”模式，推动盘锦港由运输港向贸易港转型升级。

　　加快辽东湾新区开发开放。按照“前港—中区—后城”发展模式，积极引入全国知名的专业投资公司，推进新区整体开发运营。壮大产业支撑，确保京粮等20个重点项目开工，北燃光亮油石蜡等20个项目竣工，力争再引进50个项目，实施42项基础设施建设工程。新区固定资产投资增长30%以上。

　　狠抓重点园区建设。探索建立“管委会+公司”运营模式，引导关联产业整合，加速主导产业集聚，促进各重点园区协同错位发展。完善园区基础设施，推进“四不”园区建设。各重点园区实现固定资产投资300亿元，主要经济指标持续走在辽宁沿海经济带前列。

　　扩大对外贸易。切实在开放的范围和层次上，在开放的思想观念、结构布局、体制机制上，进一步优化拓展。加大走出去力度，推进石油天然气装备制造等重点领域国际合作。更加注重提升出口质量和附加值，狠抓出口基地建设，引进和培育外贸综合服务企业，形成新的出口增量。

　　第五，千方百计抓创新创业

　　构建技术创新体系。强化科技创新，深化“市院”战略合作，推动大连理工大学盘锦校区产业技术研究院、中科院大连化物所产业技术研究院等科研院所在服务主导产业发展上实现突破，力争再引进研究院5家。引导企业加快创新平台和创新联盟建设，形成高新技术孵化基地和创新驱动发展高地，争创国家级高新区。培育一批具有创新能力的排头兵企业，年内新增小巨人企业30家、高新技术企业20家、创新型领军企业5家，全社会研发经费支出占地区生产总值比重达到2.2%。

　　促进科技成果转化。贯彻落实《促进科技成果转化和技术转移实施意见》，打开科技成果与产业对接通道，搭建科技成果发布和转化平台，加快优秀科技成果就地转移转化。全年开展核心技术攻关20项。

　　推进人才队伍建设。深入落实科技创新十条政策，开展人才工作管理服务提升年活动，突出培育和引进“高精尖”型人才。全面落实大连理工大学盘锦校区毕业生留盘就业创业优惠政策，将政策覆盖面扩展到“双一流”院校毕业生和急需紧缺型人才。培养造就一支爱家乡、讲奉献的企业家队伍、工匠队伍和劳动者大军。

　　推动大众创业万众创新。推进国家知识产权试点市建设，引导金融机构加快开展知识产权质押融资业务。鼓励大企业和科研院所、高校设立专业化众创载体，新建众创空间2万平方米以上。

　　第六，齐心协力抓乡村振兴

　　大力发展县域经济。遵循生产、生活、生态深度融合原则，坚持产业、文化、旅游、社区“四位一体”，促进县域一二三产业融合发展、县域镇域和村级集体经济整体发力、美丽乡村和特色小镇同步建设。集中打造一批产业集聚、功能复合、连城带村的特色小镇。因地制宜建设乡镇、涉农街道产业园区，加快形成“一镇一区”、“一镇一业”、“一村一品”的发展格局。

　　持续打造美丽乡村升级版。健全城乡融合发展体制机制和政策体系，清除阻碍要素下乡的各种障碍，实施市民下乡、能人回乡、企业兴乡“三乡工程”，发展“美丽乡村+”产业体系，推动以工补农、以城带乡、城乡融合发展。强化镇村规划和乡村设计，不断补齐农村基础设施短板，新建50个园林新村，积极推动“厕所革命”。

　　释放农村发展活力。深化农村集体产权制度改革，全面启动清产核资工作。抓好农村承包地“三权分置”改革，落实农民闲置宅基地和闲置农房政策，促进农村土地等资源优势转化为发展优势。完善县镇财政体制，支持合作社等集体经济发展，壮大镇村集体经济实力。破除束缚农民手脚的不合理限制和歧视，支持和鼓励农民就业创业，促进农民增收富裕。

　　强化农村治理。坚持走乡村善治之路，健全自治、法治、德治相结合的乡村治理体系，确保乡村社会充满活力、和谐有序。着力传承发展农耕文明，持续提升农民精神风貌和乡村文明程度。加强基层干部队伍建设和农村专业人才支撑，造就一支懂农业、爱农村、爱农民的“三农”工作队伍。

　　第七，凝心聚力抓国际化现代化城市建设

　　加强规划引领。全面落实《城市总体规划》，做好主体功能区规划修编，增强城乡规划的科学性、前瞻性、权威性。健全完善规划管理体系，实现城乡统筹、全域覆盖。

　　完善城市功能。推进阜盘铁路建设、疏港铁路客运化改造和中盘线改造等工程，确保东外环全线通车，做好京哈高速盘锦段扩容改造前期工作。抓好交通国际化提升工程，完成全域客运公交一体化改造，推进盘锦站、辽东湾等4个客运枢纽站建设，完善镇村客运公交站点，实现客运零距离换乘和货运无缝化衔接，创建全国道路综合运输服务示范市和全国公交都市。支持老城区综合改造提升。抓好城市街路、建筑物立面、天际线、色彩等综合设计，塑造独具特色的城市魅力。

　　强化城市管理。常态化开展“多城联创”，构建全国文明城市、国家卫生城市、国家园林城市长效管理机制。提升智慧城市建设水平，综合推进交通、旅游、医疗、社区等领域智慧系统开发应用，提高城市管理精细化、智慧化水平，迈进指尖上的城市服务新时代。着力建设网上盘锦，组建大数据公司并投入运营，为政府、企业和市民提供优质服务，带动数字经济和大数据产业加快发展。

　　第八，久久为功抓生态环境建设

　　推动形成绿色发展方式和生活方式。开展创建节约型机关、绿色家庭、绿色学校、绿色社区和绿色出行等行动，倡导简约适度、绿色低碳的生活方式。划定并严守生态保护红线、永久基本农田、城镇开发边界三条控制线。

　　加大生态系统保护力度。全面落实《盘锦市国家生态文明建设示范市规划》，持续开展国家生态文明示范县区创建。严格执行《盘锦市湿地保护条例》，做好自然保护区总体规划修编工作，实施封闭管理区、生态旅游区、生态修复区分区管理，积极争取国家生态效益补偿政策，开展滨海湿地生态修复，培育一批专门从事生态保护修复的专业化企业，恢复湿地生物多样性。启动全域绿化行动，引导各方面资金投入，植树造林200万株。

　　着力解决突出环境问题。坚决打赢污染防治攻坚战，实施蓝天、碧水、净土工程，使主要污染物排放总量大幅减少，生态环境质量总体改善。狠抓淘汰小锅炉和黄标车等工作，继续保持环境空气质量全省领先优势。全面推行“河（湖）长制”，实施大辽河、鸭子河、月牙河等河流治理工程，加强水系联通建设，确保流域和近岸海域水质稳中趋好。持续推进畜禽养殖废弃物处理和资源化利用，加强农村面源污染治理。切实抓好中央环保督察和国家海洋督察反馈意见的整改落实。

　　改革生态环境监管体制。构建政府为主导、企业为主体、社会组织和公众共同参与的环境治理和生态保护体系，完善网格化、智慧化环境监管体系。推进实施《健全生态保护补偿机制的实施意见》，推动形成陆海统筹的生态系统保护修复和污染防治区域联动机制。健全生态文明绩效考核机制，实行领导干部自然资源资产离任审计、生态环境损害责任终生追究制度，坚决制止和惩处破坏生态环境行为。

　　各位代表，提交本次大会审议的《盘锦市中心城区绿地划定方案》，是经过专家反复论证，由市委常委会讨论通过的。规划城区绿地，是我市生态文明建设的一项重大举措，功在当代、利在千秋。请大会予以审议。

　　第九，倾情倾力抓保障和改善民生

　　做实惠民之举。既尽力而为，又量力而行，加大投入，精准施策，突出办好十件重点民生实事，一件事情接着一件事情办，一年接着一年干。

　　切实提高就业质量。注重解决结构性就业矛盾，加大职业技能培训力度，促进高校毕业生、农民工多渠道创业就业，城镇新增就业1.8万人，城镇登记失业率控制在3%以内，零就业家庭保持动态为零。

　　织密扎牢社会保障网。继续实施全民参保计划。稳步提高企业职工退休人员养老待遇水平。扩大跨省直接结算定点医疗机构范围。扎实推进精准帮扶，保持年度贫困人口动态为零。加大居家和社区养老工作力度，创建全国养老服务改革试点市。

　　优先发展教育事业。加大教育投入力度。提高公办和普惠性民办幼儿园覆盖率。推进双台子区通过全国义务教育发展基本均衡区验收，促进义务教育均衡发展。在全省率先实施高中阶段学校考试招生制度改革。深化技术技能人才双元培育改革试点，推动盘锦职业技术学院“双高”建设。支持大连理工大学盘锦校区“双一流”建设。全面加强师德师风建设，严肃查处违规补课行为，切实解决中小学生课外负担重等突出问题。

　　推动文化事业和文化产业发展。发挥辽河口文化的引领作用，把地域文化与城市建设、乡村振兴结合起来，彰显盘锦文化特色。开展国家文化消费试点市建设，完成国家公共文化服务体系示范区创建任务。以辽河国际艺术区为龙头，建设文化创意、文化会展、特色文化三大产业基地，促进国际人文交流。深入实施文化惠民工程，丰富群众性文化活动。

　　推进健康盘锦建设。贯彻落实《“健康盘锦2030”行动纲要》，抓好省级健康城市试点和健康村镇建设，提高全民健康意识和健康素养。深化公立医院改革，试行“三保合一”管理体制，推行“三医联动”模式，扩大医保按疾病分组支付覆盖面。完成乡镇卫生院和社区卫生服务中心标准化建设，加快分级诊疗制度建设，实现基层医疗机构医联体全覆盖。积极创建食品药品安全城市。筹办好第13届省运会。

　　第十，持续用力抓依法治市

　　全面推进依法行政。自觉接受市人大及其常委会法律监督、工作监督和市政协民主监督，认真办理人大代表建议和政协委员提案。支持工会、共青团、妇联等群团组织更好履行职能。创新执法体制，完善执法程序，推进综合执法，严格执法责任，建立权责统一、权威高效的依法行政体制；依法全面履行政府职能，坚决纠正不作为、乱作为，坚决惩处懒政、怠政和失职、渎职行为；健全依法决策机制，强化对行政权力的制约和监督，全面推进政务公开，做好政府立法、行政复议工作，加快建设法治政府。健全完善公共法律服务体系，深入实施“七五”普法规划，增强全民法治观念，推动法治社会建设。

　　持续打造平安盘锦。深化“1+8”改革，推动社会治理重心向基层下移。坚持和完善安全稳定常态化调度评估机制，坚决遏制重特大安全事故。开展信访矛盾减存控增攻坚行动，有效化解信访矛盾纠纷和信访积案。深化公安执法规范化建设，推进“雪亮工程”，完善立体化信息化社会治安防控体系。着力解决网上虚假信息诈骗、倒卖个人信息等突出问题，严厉打击网络犯罪。蝉联全国社会治安综合治理优秀市。

　　全面加强国防和后备力量建设，深化国防教育，支持驻盘部队建设，深入开展双拥共建工作。继续做好民族宗教、外事侨务、气象地震、防灾减灾、邮政通信、老龄慈善、红十字会、质监物价、统计档案、机关事务管理等工作。

　　各位代表，实现中华民族伟大复兴的中国梦，建设国际化中等发达城市，赋予了我们这一代人光荣、艰巨、神圣的历史使命。

　　我们要坚持从严治政。筑牢“四个意识”，认真贯彻落实《关于坚决维护以习近平同志为核心的党中央权威和集中统一领导的若干规定》，自觉用习近平新时代中国特色社会主义思想武装头脑、指导实践、推动工作。把全面从严治党的要求贯穿政府各项工作始终，落实管党治党主体责任，公正用权、依法用权，营造风清气正的良好政治生态。

　　我们要坚持廉洁从政。认真学习贯彻习近平总书记关于进一步纠正“四风”加强作风建设的重要批示精神，严格执行《贯彻落实中央八项规定实施细则》，落实党政机关办公用房、公务用车管理办法，重拳整治“四风”新表现，绝不让“四风”问题反弹回潮、卷土重来。落实“一岗双责”，敬畏人民、敬畏组织、敬畏法纪，坚决纠正一切损害群众利益的不正之风和腐败行为。

　　我们要坚持实干兴政。完善绩效考评体系，完善决策部署的执行、监督、考评、奖惩等工作机制，为各级干部大胆创新撑腰鼓劲，引导全市上下勇于担当、勤奋敬业、锐意进取，以永不懈怠的精神状态和一往无前的奋斗姿态，抓实抓细抓好每一项工作，向党和人民交上合格答卷。

　　我们要深入学习贯彻党的十九大精神，持续推进“两学一做”学习教育常态化制度化，开展好“不忘初心、牢记使命”主题教育，努力增强“八个本领”，着力提升政府治理能力现代化水平。

　　各位代表，新时代要有新气象，新征程要有新作为。让我们高举习近平新时代中国特色社会主义思想伟大旗帜，更加紧密地团结在以习近平同志为核心的党中央周围，在市委的坚强领导下，埋头苦干，攻坚克难，奋力谱写建设国际化中等发达城市的新篇章，为满足全市人民日益增长的美好生活需要而不懈奋斗！
